# Application of Trio-Whole Exome Sequencing in Genetic Diagnosis and Therapy in Chinese Children With Epilepsy

**DOI:** 10.3389/fnmol.2021.699574

**Published:** 2021-08-19

**Authors:** Tiejia Jiang, Jia Gao, Lihua Jiang, Lu Xu, Congying Zhao, Xiaojun Su, Yaping Shen, Weiyue Gu, Xiaohong Kong, Ying Yang, Feng Gao

**Affiliations:** ^1^Department of Neurology, The Children’s Hospital, Zhejiang University School of Medicine, National Clinical Research Center for Child Health, Hangzhou, China; ^2^Beijing Chigene Translational Medical Research Center Co., Ltd., Beijing, China

**Keywords:** epilepsy, seizure, whole-exome sequencing, copy number variation sequencing, genetic diagnosis

## Abstract

Epilepsy is one of the most common neurological disorders in pediatric patients with other underlying neurological defects. Identifying the underlying etiology is crucial for better management of the disorder. We performed trio-whole exome sequencing in 221 pediatric patients with epilepsy. Probands were divided into seizures with developmental delay/intellectual disability (DD/ID) and seizures without DD/ID groups. Pathogenic (P) or likely pathogenic (LP) variants were identified in 71/110 (64.5%) patients in the seizures with DD/ID group and 21/111 (18.9%) patients in the seizures without DD/ID group (*P* < 0.001). Eighty-seven distinct P/LP single nucleotide variants (SNVs)/insertion deletions (Indels) were detected, with 55.2% (48/87) of them being novel. All aneuploidy and P/LP copy number variants (CNVs) larger than 100 Kb were identifiable by both whole-exome sequencing and copy number variation sequencing (CNVseq) in 123 of individuals (41 pedigrees). Ten of P/LP CNVs in nine patients and one aneuploidy variant in one patient (Patient #56, #47, XXY) were identified by CNVseq. Herein, we identified seven genes (*NCL*, *SEPHS2*, *PA2G4*, *SLC35G2*, *MYO1C*, *GPR158*, and *POU3F1*) with *de novo* variants but unknown pathogenicity that were not previously associated with epilepsy. Potential effective treatment options were available for 32 patients with a P/LP variant, based on the molecular diagnosis. Genetic testing may help identify the molecular etiology of early onset epilepsy and DD/ID and further aid to choose the appropriate treatment strategy for patients.

## Introduction

Epilepsy is one of the most common neurological disorders with 50–100 million affected, and 2–4 million new cases diagnosed each year worldwide (Pitkänen et al., 2016). Epilepsy is a chronic disorder characterized by recurrent spontaneous seizures, and often begins in childhood. Repeated and refractory seizures cause decreased social participation, long-term cognitive impairment, and significantly lower quality of life (Nickels et al., 2016). A genetic basis for some forms of epilepsy was confirmed via gene mapping in families, and the specific mutations associated with epilepsy syndromes were identified in the 1990’s (Annegers et al., 1982; Scheffer and Berkovic, 1997; Myers and Mefford, 2015).

The genetic etiology of epilepsy may be monogenic, resulting from single-gene mutations. Mutations or variants in multiple genes are also important to cause epilepsy (Møller et al., 2015). Currently, epilepsy genetics can be broadly characterized into two categories: (i) genes and loci associated with primary epilepsy; and (ii) genes associated with neurological disorders where epilepsy may be one of the symptoms (Poduri and Lowenstein, 2011). High throughput sequencing technologies have contributed to explore novel epilepsy genes. To date, numerous pathogenic variants in several genes have been associated with epilepsy and seizures (Yang et al., 2019).

The development of next-generation sequencing have greatly increased our knowledge on the genetic changes occurring across the entire human genome, allowing for the rapid and efficient discovery of genes involved in many diseases. Whole-exome sequencing (WES) is a powerful tool for detecting variants, especially the single nucleotide variants (SNVs) and the small insertions and deletions (InDels). WES is intensively being applied to clinical practice due to its low cost, high diagnostic yields, and excellent advantages regarding the analysis of novel genes and their subsequent investigation.

Diagnostic genetic tests for these complex conditions are becoming increasingly important (Berg et al., 2019) as their clinical heterogeneity and molecular complexity pose a great challenge for their clinical diagnosis and subsequent treatment. In this study, we retrospectively analyzed the diagnostic yields of trio-WES in 221 pediatric patients with epilepsy of unclear etiology and explored novel possible pathogenic genes. We aimed to explore the P/LP variants in family and specifically focus on patients with developmental delay (DD)/intellectual disability (ID) or without DD/ID. Meanwhile, we also wanted to explore the treatment strategies based on molecular diagnosis. More importantly, analysis of novel epilepsy candidate genes was performed when no pathogenic mutations were clearly identified in the characterized genetic diseases. We identified several novel genes variations such as SCN1A, MECP2, and KCNT1, which were confined as pathogenic or likely pathogenic variants of epilepsy. Herein, our results suggest that the application of WES would benefit for defining epilepsy genetic factors and treatment strategies in the clinic.

## Materials and Methods

### Study Design and Sample Collection

**FIGURE 1:**
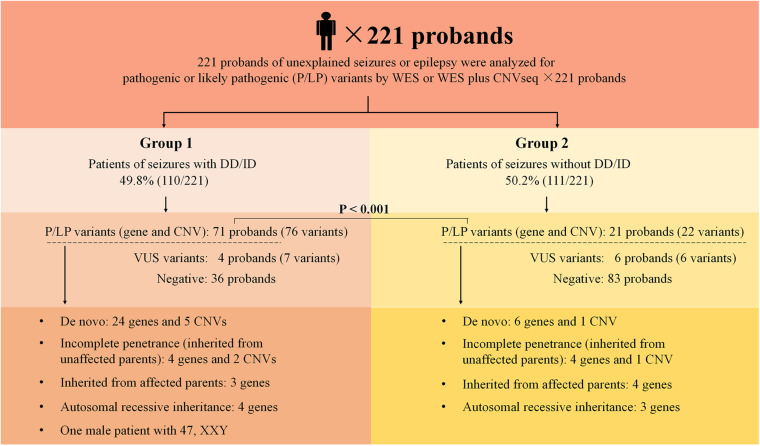
Schematic summarizing the 221 probands from non-consanguineous pedigree. Two groups were considered in this study: epilepsy with DD/ID and epilepsy without DD/ID. Pathogenic or likely pathogenic variants were categorized as: *De novo*, incomplete penetrance, inherited from affected parents, autosomal recessive inheritance. DD, developmental delay; ID, intellectual disability; P, pathogenic; LP, likely pathogenic; VUS, variant uncertain significance; CNV, copy number variant; CNVseq, CNV sequencing; WES, whole-exome sequencing.

The outline of the study design is illustrated in Figure 1. The inclusion criteria were: (1) occurrence of seizures or epilepsy before the age of 16 years-old, (2) epileptic syndromes/epileptic encephalopathy with unknown etiology, and (3) severe seizures in neonates or generalized epilepsy or intractable epilepsy in infancy with generalized tonic–clonic seizures. Patients would be excluded if the seizures were caused by non-genetic factors such as cerebral trauma, cerebral tumor, cerebral infection, cerebrovascular disorders, or diagnosed metabolic disorders. All patients underwent electroencephalogram (EEG) and magnetic resonance imaging. Epilepsy diagnoses and classifications were made by a pediatric neurologist following the criteria published by the International League Against Epilepsy. The phenotypic features of eligible patients were assessed by clinicians during the reviews of the medical records and classified according to the Human Phenotype Ontology (HPO) terms. Clinical information of the family members was obtained through face-to-face inquiries by investigators. Biological parentage was confirmed using the genomic data as described previously (Manichaikul et al., 2010).

Patients with developmental delay (DD)/intellectual disability (ID) were diagnosed by the pediatric neurologists according to the Diagnostic and Statistical Manual of Mental Disorders, Fifth Edition (DSM-5). The developmental profile of patients <36 months of age was assessed via clinical observation, the Ages and Stages Questionnaires, Third Edition (ASD-3), and the Gesell Developmental Observation-Revised (GDO-R) assessment. The Wechsler Intelligence Scale, clinical observation, and Peabody picture vocabulary tests were used for patients aged from 3 to 10 years. ID was assessed by an IQ under 70 using the Wechsler Preschool and Primary Scale of Intelligence-Fourth Edition (WPPSI-IV) for patients between the ages of 4 and 6 years, and the Wechsler Intelligence Scale for Children-Fourth Edition (WISC-IV) for patients aged over 7 years old.

### Whole-Exome Sequencing (WES)

Whole-exome sequencing and bioinformatics analyses were performed following the previously proposed guidelines (MacArthur et al., 2014; Richards et al., 2015). In brief, peripheral venous blood (2∼4 mL) was collected from the patients and their family members. The genomic DNA was extracted using the Blood genome column medium extraction kit following the manufacturer’s instructions (Kangweishiji, China). Using genomic DNA, the exonic regions and flanking splice junctions of the genome were captured using the xGen Exome Research Panel v1.0 (IDT, Coralville, IA, United States). Finally, the libraries were sequenced on an Illumina NovaSeq 6000 series sequencer with the following parameters: PE150, minimum of 11.6 million reads. The sequencing was performed by the Beijing Chigene Translational Medicine Research Center Co., Ltd., Beijing, China.

Raw data were processed using the *fastp* tool to remove the adapters and filter out the low-quality reads. The paired-end reads were performed using a Burrows-Wheeler Aligner (BWA) against the Ensembl GRCh37/hg19 human reference genome (Li and Durbin, 2010). Both SNVs and small InDels were called using the Genomic Analysis Toolkit (GATK) software (version 4.1.7) (McKenna et al., 2010). The copy number variant (CNV) calling was based on the ExomeDepth algorithm. The total read count of the sample mapped to each exon in the same batch as described previously (Plagnol et al., 2012).

Variants were annotated using an online system, developed by Chigene which contains 35 public databases, while our in-house database contains WES data from 69015 individuals (Supplementary Table 1). Candidate SNVs/small InDels were confirmed by Sanger sequencing. A small CNV (<10 kb) would be considered if the phenotype was highly related to the candidate gene located in this CNV region; these were confirmed by quantitative polymerase chain reaction (qPCR). We classified the candidate variants according to the American College of Medical Genetics and Genomics (Richards et al., 2015) and Sequence Variant Interpretation Working Group international guidelines (SVI WG)^1^.

### Copy Number Variation Sequencing (CNVseq)

Copy number Variation Sequencing (CNVseq) was performed as previously described (Gao et al., 2019). Briefly, the genomic DNA was fragmented by sonication (Covaris, United States) into 200–300 bp fragments and checked using agarose gel electrophoresis. After genomic library preparation, DNA samples were subsequently sequenced on an Illumina NovaSeq 6000 series sequencer (Illumina, San Diego, CA, United States). Raw image files were processed using BclToFastq (Illumina) for the base calling and raw data generation. The reads were then mapped to the GRCh37/hg19 human reference genome using the BWA software (Li and Durbin, 2010). Variant calling for CNVs ≥100 kb was performed using an in-house pipeline, and the candidate CNVs were filtered and detected using public CNV databases (Decipher, ClinVar, OMIM, DGV, and ClinGen). The pathogenicity of CNVs was classified according to the American College of Medical Genetics and Genomics guidelines (Riggs et al., 2020).

### Identification of Candidate Pathogenic *de novo* Variants

Variants (SNVs and InDels in coding region; canonical ± 1 or 2 splice sites) were considered to be candidate pathogenic *de novo* if they met the following criteria: (1) in patients with normal parental phenotype; (2) genotype call ratio > 0.3 and supporting read depth > 20; (3) minor allele frequency (MAF) < 0.0001 as reported in the Genome Aggregation Database (gnomAD); (4) Pathogenic variants were in the Ensembl canonical transcript.

### Statistical Analysis

Categorical data are expressed in percentage and the comparisons between the groups were analyzed using the Pearson’s Chi-square test or a two-tailed Fisher’s exact test (for *N* < 40), in which a *P*-value smaller than 0.05 was considered to be statistically significant. We performed statistical analyses using SPSS software, version 25.0 (SPSS Inc., Chicago, IL, United States).

## Results

### Participant Demographics and Phenotypes

Two hundred and twenty one of unrelated patients (96 females and 125 males) and their families were recruited from our hospital between January 2016 and November 2019. Patients were from non-consanguineous families in Southeast China. The age of seizure onset ranged from 1 day after birth to 15-years old. Sixty-six of the patients had family history of seizures. Patients were divided into two groups: the seizures with DD/ID group and the seizures without DD/ID group (Figure 1). Clinical information of the patients was summarized in Supplementary Tables 2, 3.

### Molecular Diagnosis Yields

**FIGURE 2:**
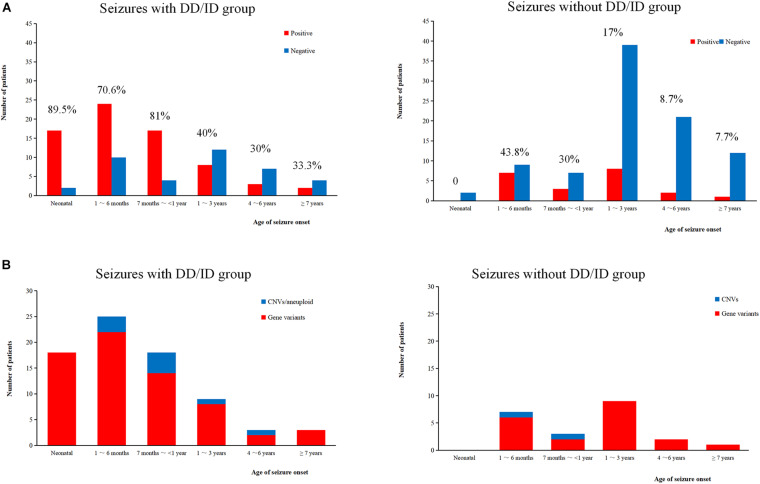
Molecular diagnosis yield in different ages. **(A)** The molecular diagnosis yield in the groups of epilepsy with DD/ID and without DD/ID during different ages of seizure onset. **(B)** The individuals containing gene variants in the groups of epilepsy with DD/ID and without DD/ID during different ages of seizure onset. The percentage in the diagram represents the diagnosis positive rate = (P + LP)/all individuals in this age.

We conducted WES to detect the epilepsy-associated gene variants. Pathogenic (P) or likely pathogenic (LP) variants were identified in 92 patients (92/221 = 41.6%), consisting of 87 distinct gene-level variants in eighty-two patients, 10 CNVs in nine patients, and one male patients with 47, XXY. These mutations were found in 71 patients in the group of seizures with DD/ID (71/110 = 64.5%) and 21 patients in the group of seizures without DD/ID group (21/111 = 18.9%), respectively (Figure 2A). The patients in the group of seizures with DD/ID had more P/LP mutations than those in the group of seizures without DD/ID (*P* < 0.001) (Figure 2B). Interestingly, the patients with seizures in DD/ID group under 1 year-old showed more P/LP variants than those of other groups. P/LP gene variants were identified in sixty patients under 1 year-old and 86.7% of the patients belong to DD/ID group. Intriguingly, 47 *de novo* variants and 29 novel variants were identified in 62 variants in the patients under 1-year-old (Table 1). Moreover, the number of *de novo* gene variants in patients with DD/ID was more than that of patients without DD/ID (Table 2). In addition, we also identified 13 variants of uncertain significance (VUS) in 4.5% (10/221) of the patients (Supplementary Table 3).

**TABLE 1:** Number of patients with P/LP gene variants.

	Patients with P/LP variants	Patients with P/LP of seizure onset <1 year variants
**Total**	92	68
**With DD/ID**	71	58
**Without DD/ID**	21	10
**Gene variations**		
**Individuals containing gene variations**	82	60
Total gene variants	87	62
No. of *de novo* variants	58	47
No. of Novel variants	48	29
**Patients of CNVs/Aneuploid**	10	8
No. of variants	11	9
No. of *de novo* variants	8	7

**TABLE 2:** Number of *de novo* and novel gene variants in the patient under 1 year-old.

	With DD/ID	Without DD/ID	Total
*De novo* variants	43	4	47
Novel variants	26	3	29

### Gene Variants

**FIGURE 3:**
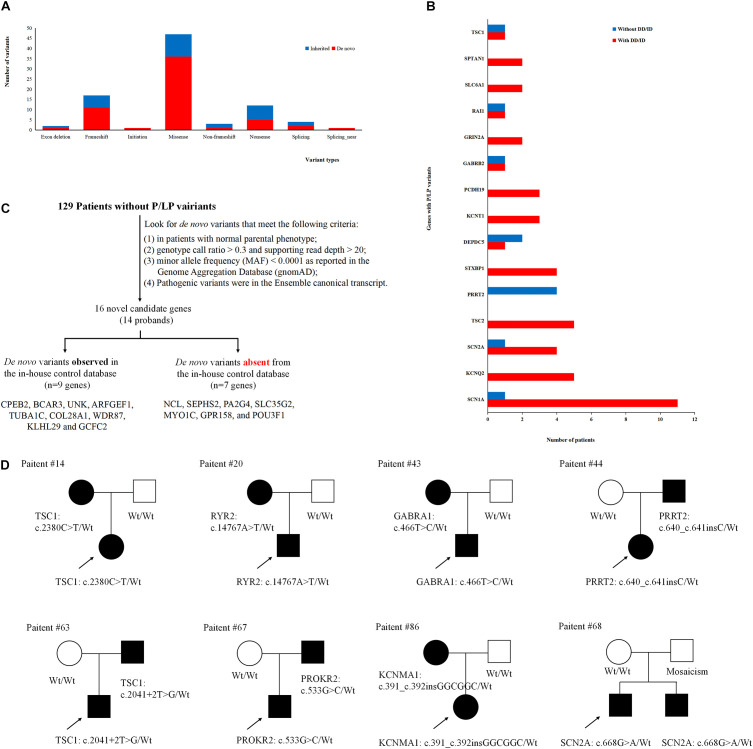
Gene variants analysis. **(A)** The number of different variant types observed in patients. **(B)** Identification procedure of candidate pathogenic *de novo* variants. **(C)** Distribution of recurrent (≥2 patients) genes with pathogenic or likely pathogenic variants in the groups of epilepsy with DD/ID and without DD/ID. **(D)** The diagram of epilepsy pedigrees. ∘ represents female; □ represents male; 🌑 and ■ represents affected individuals; arrow represents probands; wt represents wild type.

To better understand the epilepsy-associated gene variants, we categorized the gene variants as *de novo*, incomplete penetrance, inherited from affected parents, and autosomal recessive (AR) variants. We found 58 *de novo* gene variants and 48 novel variants (Table 3, Figure 3A, and Supplementary Table 2). Thirty-two genes were associated with DD/ID group. *SCN1A* was most frequently involved, followed by *KCNQ2* and *TSC2*. *PRRT2* was most frequently involved in the group of seizures without DD/ID (Figure 3B).

**TABLE 3:** Forty-eight novel pathogenic or likely pathogenic variants.

Gene	Gene NM#	Variants of nucleic acid	Variants of protein	het/hom
*ALDH7A1*	NM_001182	exon 12	/	het
*AP4M1*	NM_004722	c.1264C>T	p.R422X	het
*ARID1B*	NM_020732	c.1910_c.1911 delGG	p.R637Ifs*15	het
*ATM*	NM_000051	c.7878_c.7882del TTATA	p.A2626Afs*28	het
*DEPDC5*	NM_001242896	c.562+1G>T	/	het
	NM_001242896	c.2731G>T	p.E911X	het
	NM_001242896	c.484-1_c.485delGGT	p.V162Gfs*18	het
*DLG3*	NM_021120	c.1861C>T	p.R621W	het
*EEF1A2*	NM_001958	c.289G>A	p.D97N	het
*GABRA1*	NM_001127644	c.466T>C	p.Y156H	het
*GABRB2*	NM_021911	c.946G>A	p.V316I	het
	NM_021911	c.486G>T	p.M162I	het
*GRIN2A*	NM_001134407	c.1965delA	p.Q655Qfs*8	het
	NM_001134407	c.2389delinsCAG	p.T797Qfs*12	het
*KCNMA1*	NM_001271520	c.391_c.392ins GGCGGC	p.L131delinsRRL	het
*KCNQ2*	NM_004518	c.836G>T	p.G279V	het
	NM_172107	c.485A>G	p.K162R	het
	NM_172107	c.504_c.505delCT	p.F168Lfs*4	het
*MBD5*	NM_018328	c.1628C>T	p.T543I	het
*NPRL2*	NM_006545	c.673C>T	p.Q225X	het
*PCDH19*	NM_001105243	c.497dupA	p.Y166X	het
	NM_001184880	c.470A>G	p.D157G	het
*PLA2G6*	NM_003560	c.127C>T	p.Q43X	hom
*PRRT2*	NM_001256443	c.489delG	p.Q163Qfs*13	het
*RAI1*	NM_030665	c.3301C>T	p.P1101S	het
	NM_030665	c.5254_c.5266 delGGGAAGCC CCCCA	p.G1752Gfs*94	het
*RYR2*	NM_001035	c.14767A>T	p.M4923L	het
*SCN1A*	NM_001202435	c.724C>T	p.Q242X	het
	NM_001165963	c.2791C>T	p.R931C	het
	NM_001202435	c.603-2A>T	/	het
	NM_001202435	c.4476+3_c.4476 +8 delAAGTAT	/	het
	NM_001165963	c.632delA	p.N211Mfs*5	het
	NM_001202435	c.479C>A	p.T160N	het
	NM_001202435	exon:26-27	/	het
*SCN2A*	NM_021007	c.4959G>C	p.L1653F	het
	NM_021007	c.4823-2A>C	NA	het
*SLC13A5*	NM_177550	c.202C>T	p.P68S	het
	NM_177550	c.429_c.437 delGCCGTGGTT	p.A143_A146 delinsA	het
*SLC6A1*	NM_003042	c.1367G>A	p.S456N	het
	NM_003042	c.1348_c.1349de lTT	p.F450Xfs*1	het
*SPTAN1*	NM_001130438	c.1595A>G	p.K532R	het
	NM_001130438	c.6614_c.66 16delAGG	p.Q2205_E2206 delinsQ	het
*STXBP1*	NM_003165	c.814delG	p.G272Gfs*5	het
	NM_003165	c.990dupG	p.L331Afs*21	het
	NM_003165	c.1A>T	p.M1L	het
*TDP2*	NM_016614	c.650delG	p.G217Efs*7	hom
*TSC1*	NM_000368	c.2041+2T>G	/	het
*TSC2*	NM_000548	c.3023_c.3038del TGGCCCAGG CTGACGA	p.V1008Vfs*3	het

It is interesting to explore the novel gene variants in the 129 epilepsy patients without P/LP variants. So we re-analyzed the data and selected 16 novel candidate genes from 14 individuals (Figure 3C and Supplementary Table 4); however, nine of the genes contain several *de novo* variants in the in-house control database, suggesting that these nine genes cannot be evaluated as the *de novo* genes associated with epilepsy. *De novo* variants in the other seven genes (*NCL*, *SEPHS2*, *PA2G4*, *SLC35G2*, *MYO1C*, *GPR158*, and *POU3F1*) were not found in the in-house control database. In addition, we found that *GPR158* and *POU3F1* are highly expressed in the nervous system according to the Genotype-Tissue Expression (GTEx) database^2^. It suggests that these seven genes may be related to epilepsy, while the functions of them need to be further confirmed.

Moreover, we also acquired seven variants from affected parent families, including *TSC1*, *PRRT2*, *TSC1*, *PROKR2*, *RYR2*, *GABRA1*, and *KCNMA1* genes (Figure 3D). Interestingly, a *de novo* variant, *SCN2A* c.668G>A was detected in patient #68 with epileptic encephalitis. Her brother also had *SCN2A* c.668G>A variant and showed hand clenching accompanied by slight shaking and up rolling of eyeballs. Then, we conducted ultra-deep sequencing (average deep: 20000×) and detected the *SCN2A*: c.668G>A variant in father’s oral formulas, urine and seminal fluid. The results showed that the mosaicism percentage of oral formulas, urine and seminal fluid were 13.14, 12.7, and 23.26%, respectively. We confirmed that SCN2A c.668G>A variant was paternal germ line mosaicism (Figure 3D and Supplementary Table 2).

### CNVs by WES and CNVseq

**FIGURE 4:**
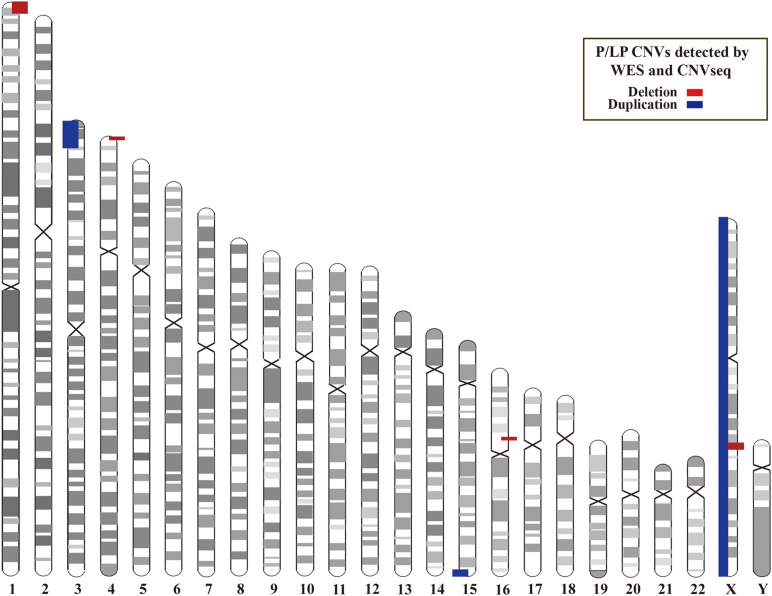
Locations of P/LP CNVs identified by WES and/or CNVseq. CNV, copy number variant; CNVseq, CNV sequencing; WES, whole-exome sequencing.

In addition, we investigated CNVs in 123 of individuals (41 pedigrees). All aneuploidy and P/LP CNVs (>100 kb in size) were identifiable by WES and CNVseq (Figures 1, 4). Ten P/LP CNVs in nine patients and one aneuploidy variant in one patient (Patient #56, #47, XXY) were identified by CNVseq. Three CNVs were located in chromosome 16 and belong to 16p11.2 deletion syndrome. Two CNVs were duplications and eight CNVs were deletions, ranging from 411 to 12 Mb (Table 4 and Supplementary Tables 5, 6). Patient #73 carried two *de novo* CNVs, which manifests as developmental delay and seizures. One of the CNVs was a deletion and located in Chr4, including *ZNF141*, *PIGG*, *PDE6B*, and *CPLX1* genes; another CNV was a duplication variant and located in Chr15, involving *MEF2A*, *ADAMTS17*, *CERS3*, *LINS*, *ALDH1A3*, and *CHSY1* genes. It was demonstrated that *CPLX1* gene functional aberration caused severe infantile myoclonic epilepsy and ID (Redler et al., 2017).

**TABLE 4:** Pathogenic/likely pathogenic CNVs identified by WES and CNVseq in forty-one pedigrees.

Patient ID	ASD	DD/ID	Position (hg19)	Size	Type	Inheritance	Associated Genetic Syndrome (OMIM disease ID)	Interpretation
26	N	N	chr4:76393-1613145	1.5M	deletion	*De novo*	/	LP
41	N	P	chr3:60001-12282678	12M	duplication	*De novo*	/	P
48	N	P	chr1:10001-4651608	4.6M	deletion	*De novo*	1p36 deletion syndrome (OMIM:#607872)	P
73	N	P	chr4:53382-964860	911Kb	deletion	*De novo*	/	LP
	/	/	chr15:100214597-102284775	2M	duplication	*De novo*	/	LP
76	N	P	chrX:96603114-99663595	3M	deletion	*De novo*	/	P
81	P	P	chr16:29468170-30301199	833Kb	deletion	*De novo*	16p11.2 deletion syndrome (OMIM:#611913)	LP
58	N	P	chr16:29594293-30171789	577Kb	deletion	paternal	16p11.2 deletion syndrome (OMIM:#611913)	LP
78	N	N	chr16:29571179-30189789	618Kb	deletion	paternal	16p11.2 deletion syndrome (OMIM:#611913)	LP
87	N	P	chr16:21964744-22376335	411Kb	deletion	paternal	16p12.1 deletion syndrome (OMIM:#136570)	LP

### Therapeutic Implications

There were specific therapeutic recommendations for 10 genes with P/LP variants in the current cohort, including *ALDH7A1* (*n* = 1), *DEPDC5* (*n* = 3), *GRIN2A* (*n* = 2), *KCNQ2* (*n* = 5), *SCN1A* (*n* = 12), *SCN2A* (*n* = 5), *SLC2A1* (*n* = 1), *SLC6A1* (*n* = 2), *TSC1* (*n* = 2), and *TSC2* (*n* = 5). In this study, 32 patients were applicable drug selection based on molecular diagnosis. For example, the patient #24 was a 13 years-old female with mild ID. Prior to the genetic testing referral, she kept monthly seizures despite treatment with sodium valproate, levetiracetam, and lamotrigine. Genetic test revealed a missense variant in *SLC2A1* (c.997C>T, p.R333W), which cause the GLUT1 deficiency syndrome. Then a ketogenic diet was initiated based on the genetic results. Surprisingly, the patient kept seizure-free developmental improvement (cognitive and behavioral) after treatment. She has subsequently been tapered off all of the anti-epileptic drugs. For patients with the *SCN1A* gene mutations, a combination of VPA and TMP improved the seizures effectively and the whole treatment process should not use sodium channel blockers. In addition, vigabatrin treatment decreased the seizure frequency and improved EEG in four of patients with *TSC1* or *TSC2* gene mutations. Oxcarbazepine was effective for five of patients with *KCNQ2* gene mutations (Table 5).

**TABLE 5:** Treatment strategies of 32 cases of patients based on WES diagnosis.

Patient ID	Age of seizure onset	Diagnosis	Treatment Impact	All Treatment	Prognosis	Gene	Variants of nucleic acid	Variants of protein
6	1 year 1 month	FE	Treated with VB6	VPA, VB6	No seizures in the last years	*ALDH7A1*	c.1061A>G	p.Y354C
						*ALDH7A1*	exon 12	/
10	1 month 4 days	FE	Treated with KD, improved in seizure frequency	OXC, TPM, VPA, LEV, LTG, KD	4 seizures in the last years	*DEPDC5*	c.562+1G>T	/
19	3 years 2 months	EE (EAS)	Treated with ACTH and LEV, decreased epileptic discharges during sleep	VPA, ACTH, LEV	5 seizures in the last years	*GRIN2A*	c.1965delA	p.Q655Qfs*8
77	3 years 10 months	EE (EAS)	Treated with LEV, decreased epileptic discharges during sleep	VPA, LEV	No seizures in the last years	*GRIN2A*	c.2389delinsCAG	p.T797Qfs*12
23	7 days	EE (OS)	Treated with OXC, improved the seizure frequency	VPA, TMP, OXC	1 seizures in the last years	*KCNQ2*	c.587C>T	p.A196V
34	1 day	EE (OS)	Treated with OXC, no change in seizure frequency	NZP, LEV, OXC	1–3 seizures per month	*KCNQ2*	c.836G>T	p.G279V
40	2 day	EE (OS)	Treated with OXC, improved the seizure frequency	PB, OXC, TMP, OXC	No seizures in the last 5 months	*KCNQ2*	c.881C>T	p.A294V
80	3 days	EE	Treated with OXC, improved the seizure frequency	TMP, OXC	No seizures in the last 3 months	*KCNQ2*	c.485A>G	p.K162R
89	9 days	EE	Treated with OXC, improved the seizure frequency	VPA, TMP, OXC	Seizures almost every months	*KCNQ2*	c.504_c.505delCT	p.F168Lfs*4
5	7 months	EE (DS or DS-like)	Avoiding sodium channel blockers and Change from LEV to VPA, TMP	LEV, CZP, VPA, TPM	Seizures 1–2 times a year, mostly heat-related	*SCN1A*	c.724C>T	p.Q242X
7	7 months	EE (DS or DS-like)	Avoiding sodium channel blockers and started Valproic acid early	VPA, TPM	Seizures 1–2 times a year, mostly heat-related	*SCN1A*	c.1198A>C	p.M400L
33	7 months	EE (DS or DS-like)	Avoiding sodium channel blockers and Change from LEV to VPA, TMP	LEV, TMP, VPA	4 seizures in the last years, heat-related	*SCN1A*	c.603-2A > T	/
35	7 months	EE (DS or DS-like)	Avoiding sodium channel blockers and unnecessary medical investigations	VPA, TMP	3 seizures in the last years, heat-related	*SCN1A*	c.4476+3_c.4476+8 delAAGTAT	/
38	1 months 25 days	EE	Change from OXC to VPA, improved the seizure frequency	OXC, VPA	No seizures in the last 3 months	*SCN1A*	c.677C>T	p.T226M
39	5 months	EE (DS or DS-like)	Avoiding sodium channel blockers	CZP, TMP, LEV	5 seizures in the last years, heat-related	*SCN1A*	c.632delA	p.N211Mfs*5
42	8 months	GE	Avoiding sodium channel blockers and started Valproic acid early	VPA, LEV	No seizures in the last 3 months	*SCN1A*	c.695G>T	p.G232V
53	8 months	EE (DS or DS-like)	Avoiding sodium channel blockers	VPA, LEV	4 seizures in the last years, heat-related	*SCN1A*	c.2134C>T	p.R712X
65	5 months	EE (DS or DS-like)	Avoiding sodium channel blockers and started Valproic acid early	VPA, LEV	No seizures in the last 3 months	*SCN1A*	c.5339T>G	p.M1780R
72	5 months	EE (DS or DS-like)	Change from OXC to VPA, improved the seizure frequency	OXC, VPA	4 seizures in the last years, mostly heat-related	*SCN1A*	c.479C>A	p.T160N
92	8 months	EE	Avoiding sodium channel blockers and started Valproic acid early	VPA, TMP	2 seizures in the last years, heat-related	*SCN1A*	exon:26-27	/
30	19 days	EE (OS)	Treated with LCS, no improvement in seizure frequency	VPA, NZP, TMP, LCS	Seizures almost every month	*SCN2A*	c.4959G>C	p.L1653F
52	1 years 9 months	FE	Treated with LCS, improved the seizure frequency	OXC, LCS	No seizures in the last years	*SCN2A*	c.4823-2A>C	NA
54	1 days	EE	Treated with LCS, improved the seizure frequency	OXC, TMP, LCS	Seizures almost every weeks	*SCN2A*	c.640T>C	p.S214P
24	5 months	EE, GULT1-DS	Treatment with KD, seizure-free and significant improvement in development, and significant progress in cognitive and behavioral development	VPA, LEV, LTG, KD	No seizures in the last 3 years	*SLC2A1*	c.997C>T	p.R333W
61	4 years	EE (DOOSE)	Treated with KD, improved the seizure frequency	LEV, NZP, KD	No seizures in the last years	*SLC6A1*	c.1348_c.1349delTT	p.F450Xfs*1
14	11 months	EE, TSC	Influenced choice of future treatment	VPA, CZP, TMP, LEV	3 seizures in the last years	*TSC1*	c.2380C>T	p.Q794X
63	1 years 3 months	FE, TSC	Influenced choice of future treatment	CBZ	No seizures in the last 3 months	*TSC1*	c.2041+2T>G	/
3	9 months	FE, TSC	Treated with VGB and Rapamycin, improved the seizure frequency and EEG	VPA, VGB, Rapamycin	3–5 seizures per week	*TSC2*	c.3023_c.3038del TGGCCCAGG CTGACGA	p.V1008Vfs*3
9	1 years	FE, TSC	Influenced choice of future treatment	VPA, TMP	No seizures in the last years	*TSC2*	c.4925G>A	p.G1642D
16	7 months	EE (West), TCS	Treated with VGB, improved the seizure frequency and EEG	ACTH, Prednisone, VPA, TMP, VGB	5 seizures in the last years	*TSC2*	c.3608C>G	p.T1203R
70	5 months	FE, TSC	Treated with VGB, improved the seizure frequency and EEG	ACTH, Prednisone, VPA, LCS, VGB	Seizures almost every days	*TSC2*	c.4868C>T	p.T1623I
83	11 months	EE (WEST)	Treated with VGB and LEV, improved the seizure frequency and EEG	Prednisone, VPA, VGB, LEV	Seizures almost every weeks	*TSC2*	c.1831C>T	p.R611W

## Discussion

Genetic factors were estimated to play a role in 70∼80% of epilepsy cases, especially in children and neonates (Hildebrand et al., 2013). Several studies have focused on the application of next-generation sequencing as a diagnostic tool for epilepsy (Veeramah et al., 2013; Dyment et al., 2015; Parrini et al., 2017). Recent cohort studies suggested that the diagnostic yield of WES varies from 23 to 42% in patients with epilepsy (Helbig et al., 2016; McTague et al., 2016; Costain et al., 2019; Snoeijen-Schouwenaars et al., 2019; Yang et al., 2019; Johannesen et al., 2020; Rochtus et al., 2020). In these studies, the phenotypes of patients varied widely, the inclusion and exclusion criteria of patients were also not consistent, and the pathogenic genes/pathways might be different. In the present study, the overall diagnostic yield was 41.6%. Further, we also found the diagnostic yield of the seizures with the DD/ID group to be higher than that in previous studies, especially in seizure onset under 1-year-old (∼78.4%) (Trump et al., 2016; Yang et al., 2019). It may be attributed to the following reasons. Firstly, our study analyzed SNVs, InDels, and CNVs, which can lead to a higher diagnostic yield. Secondly, the non-randomized selection/hospital-enrichment of the patients may lead to sampling bias. Pediatricians were likely to have subjective preference in the selection of patients with DD/ID for clinical genetic testing as it is easier to discover disease-related P/LP variants. Thirdly, for some patients in the seizures without DD/ID group, the patients under 4 years old might develop to ID later.

In the present study, thirteen of the patients carried P/LP variants that are inherited from unaffected parents (Supplementary Table 2), 53.8% (7/13) of them had autism spectrum disorder (ASD)/DD/ID (Figure 1). The unaffected phenotype of carriers was likely due to the incomplete penetrance, which was previously reported for the six genes: *DEPDC5*, *SCN1A*, *PCDH19*, *PRRT2*, *GRIN2A*, and *NPRL2*; 16p11.2 deletion and 16p12.1 microdeletion syndrome suggested that other modifier gene(s), as well as epigenetic or environmental factors, modulate the phenotype (Weiss et al., 2008; Girirajan et al., 2010; Dimova et al., 2012; Heron et al., 2012; Ishida et al., 2013; Lesca et al., 2013; Meng et al., 2015; Ricos et al., 2016). For example, the penetrance of *DEPDC5* variants with different forms of focal epilepsy was incomplete, varying from 50 to 82% (Ishida et al., 2013; Ricos et al., 2016). In this study, the *DEPDC5* gene variants were null variants (c.562+1G>T, c.2731G>T, c.484-1_c.485delGGT) carried by three patients with focal epilepsy (Patient #10, #46, and #84). Only patient #10 had epilepsy with DD. Actually the patient #10 carried two *DEPDC5* variants, c.562+1G>T and c.2507A>G (p.Y836C), the latter was inherited from his asymptomatic father. We can’t confirm the *DEPDC5* gene with an AR inheritance in our local database. So this bi-allelic defect may exacerbate the clinical symptoms and further studies are required to confirm the functions.

Some studies defined that therapeutic outcomes of epilepsy were mostly based on the effect of protein function, clinical observation, and literature reports (Schoonjans et al., 2017; Yang et al., 2019; Johannesen et al., 2020). Herein, we reported the choices of therapeutic intervention in 32 of patients were affected based on the genetic diagnosis and the symptoms of some patients were improved effectively (Table 5). As some types of epilepsies responded to particular antiepileptic medications, personalized therapeutic strategies will be the best choice of epilepsy therapy. The top three most frequently mutated genes were the same as reported in this cohorts, including *SCN1A*, *KCNQ2*, and *TSC2* (Yang et al., 2019). In addition, we also found that *PRRT2* heterogeneous variant was the most frequent mutated gene in the group of seizures without DD/ID. Moreover, we detected three cases with 16p11.2 deletion (includes *PRRT2* gene). Two of the patients (#58 and #81) were with DD/ID and one patient (#78) hasn’t shown DD/ID (<4 years-old). Our results are consistent with the previous reported (Ebrahimi-Fakhari et al., 1993; Termsarasab et al., 2014). Ebrahimi-Fakhari et al. (1993) reported that the patients with *PRRT2* heterogenous variants commonly exhibited epilepsy and paroxysmal movement disorders (PRRT2-associated paroxysmal movement disorders, RRT2-PxMD) without intellectual delay. While the individuals with 16p11.2 deletion, or with rare bi-allelic *PRRT2* pathogenic variants exhibited DD/ID or ASD. Meanwhile, we identified seven genes with *de novo* variants in pathogenically uncertain patients, which included the previously reported *GPR158* (OMIM: 614573) gene. *GPR158* gene is related to seizures (Elmariah et al., 2014) and highly expressed in the nervous system. It may be a promising epilepsy candidate gene. The function of the other six genes (*NCL*, *SEPHS2*, *PA2G4*, *SLC35G2*, *MYO1C*, and *POU3F1*) is currently unknown in the nervous system and the gene variations were observed in only one individual. The functions of these genes need to be further confirmed. Briefly, WES could help physicians identify epilepsy-associated genes in early onset patients and further provide effective treatment in clinic and improve patients’ life quality.

In fact, this study still had several limitations. Firstly, our data revealed several *de novo* SNVs/InDels; however, mosaicism was not confirmed. Secondly, we didn’t identify the plausible causal mutations in more than half of the patients. It indicates that these patients may not be an aggregate of simple Mendelian disorders and therefore require further powerful tools to evaluate the disease elucidation. Thirdly, in our study, although we identified seven potential candidate genes related to epilepsy disease, there is currently not enough evidence to support their pathogenicity. Therefore, a more comprehensive testing tool and further genetic studies with larger cohorts are required to fully elucidate the underlying etiology. Meanwhile, functional tests are urgent for assessing the epilepsy-associated genes.

In conclusion, our study demonstrates that the simultaneous analysis of SNVs, InDels, and CNVs based on NGS data can provide a high diagnostic yield for epilepsy, especially for patients with DD/ID, age of seizure onset under 1-year-old. We further demonstrate the potential of genetic diagnosis impacts on choosing the optimal treatment strategy for these patients.

## Data Availability Statement

The original contributions presented in the study are included in the article. Data on patients cannot be made fully accessible in accordance with local research ethics protocols. Further inquiries can be directed to the corresponding author/s.

## Ethics Statement

The studies involving human participants were reviewed and approved by the Ethics Committee of the Children’s Hospital, Zhejiang University School of Medicine. Written informed consent to participate in this study was provided by the participants’ legal guardian/next of kin.

## Author Contributions

TJ, JG, LJ, and FG designed the data collection instruments, collected the data, and carried out the initial analyses. TJ, YS, YY, and FG reviewed and revised the manuscript. LX, CZ, XS, WG, and XK conceptualized the study and coordinated and supervised data collection. All authors approved the final manuscript as submitted and agreed to be accountable for all aspects of the work.

## Conflict of Interest

WG, XK, YY was employed by company Beijing Chigene Translational Medical Research Center Co., Ltd. The remaining authors declare that the research was conducted in the absence of any commercial or financial relationships that could be construed as a potential conflict of interest.

## Publisher’s Note

All claims expressed in this article are solely those of the authors and do not necessarily represent those of their affiliated organizations, or those of the publisher, the editors and the reviewers. Any product that may be evaluated in this article, or claim that may be made by its manufacturer, is not guaranteed or endorsed by the publisher.

## Footnotes

1
https://www.clinicalgenome.org/working-groups/sequence-variant-interpretation/

2
https://www.gtexportal.org/home/

## Supplementary Material

Supplementary Table 1 The information of the public databases used in Chigene database.

Supplementary Table 2 Clinical and molecular features of positive patients with positive genetic diagnosis. M, male; F, female; Y, year(s); M, month(s); D, day(s). Diagnosis: BFIS, benign familial infantile seizures; CCHD, critical congenital heart disease; CPVT, catecholaminergic polymorphic ventricular tachycardia; DOOSE, Doose Syndrome; DS or DS-like, Dravet syndrome or Dravet syndrome-like; EAS, epilepsy-aphasia spectrum; EE, epileptic encephalopathy; FE, focal epilepsy; FFEVF, familial focal epilepsy with variable foci; GE, generalized epilepsy; GEFS+, genetic epilepsy with febrile seizures plus; GULT1-DS, glucose transporter type 1 deficiency syndrome; IT, immune thrombocytopenia; LGS, Lennox-Gastaut Syndrome; MMPSI, malignant migrating partial seizures of infancy; OS, Ohtahara syndrome; PKD, paroxysmal kinesigenic dyskinesia; RTT, Rett syndrome; TSC, tuberous sclerosis; WEST, West syndrome. FOS, focal onset; FS, febrile seizures; GOS, generalized onset; DD, Developmental delay: ASD, autism spectrum disorder. Treatment: ACTH, adrenocorticotropic hormone; CBZ, carbamazepine; CLB, clobazam; CZP, clonazepam; KD, ketogenic diet; LCS, lacosamide; LCT, L-carnitine; LEV, levetiracetam; LTG, lamotrigine; NZP, nitrazepam; OXC, oxcarbazepine; PA, prednisone acetate; PB, phenobarbitone; RPM, rapamycin; TPM, topiramate; VB6, vitamin B6; VGB, vigabatrin; VPA, valproate. AR, autosomal recessive; AD, autosomal dominant; XL, X-linked; XLD, X-linked dominant; XLR, X-linked recessive; P, positive; N, negative; nd, not determined.

Supplementary Table 3 Clinical and molecular features of patients with variants of uncertain significance (VUS) identify in the cohort. M, male; F, female; Y, year(s); M, month(s); D, day(s); AR, autosomal recessive; AD, autosomal dominant; P, positive; N, negative; nd, not determined.

Supplementary Table 4 Clinical and molecular features of patients with *de novo* variants identified in the negative cohort. M, male; F, female; Y, year(s); M, month(s); D, day(s); AR, autosomal recessive; AD, autosomal dominant; XLD, X-linked dominant; XLR, X-linked recessive.

Supplementary Table 5 Sequencing data quality of whole-exome sequencing and CNVseq for the patients and their parents. CNVseq, copy number variant sequencing.

Supplementary Table 6 Pathogenic/likely pathogenic CNVs identified by WES and CNVseq in the cohort. M, male; F, female.
